# Comparability of CMV DNA Extraction Methods and Validation of Viral Load

**DOI:** 10.3390/mps5010006

**Published:** 2022-01-04

**Authors:** Théophile Uwiringiyeyezu, Bouchra El Khalfi, Rachid Saile, Jamal Belhachmi, Abdelaziz Soukri

**Affiliations:** 1 Research Center of Biotechnology and Health, Laboratory of Physiopathology, Molecular Genetics and Biotechnology, Faculty of Sciences Aïn Chock, Hassan II University of Casablanca, B.P 5366 Maarif, Casablanca 20000, Morocco; bouchra.elkhalfi@gmail.com; 2 Laboratory Al Kindy of Medicals Analysis, Casablanca 20100, Morocco; jbelhachmi@gmail.com; 3 Research Center of Biotechnology and Health, Laboratory of Biology and Health, Faculty of Sciences, Ben M’sik, Hassan II University of Casablanca, B.P 5366 Maarif, Casablanca 20000, Morocco; sailerachid@yahoo.fr

**Keywords:** molecular biology, nucleic acids, polymerase chain reaction, cytomegalovirus, transplanting, graft rejection, medical analysis, extraction techniques, threshold cycle (Ct)

## Abstract

Human cytomegalovirus is a herpesvirus that has a worldwide seroprevalence of more than 60% of adults in developed countries and 90% in developing countries. Severe disabilities in newborns are characteristic of the human cytomegalovirus congenital infection, and this virus is implicated in graft rejection in transplant patients. To treat and follow-up the infection, the CMVPCR viral loads are required, and the DNA extraction step remains very important; however, the quantity, quality, and purity of extracted DNA from different biological fluids influence the results of PCR amplification, that is why for reliable results, the choice of nucleic acid extraction methods requires careful attention. **Materials and methods**: In this study, we compare 4 protocols, I (EZ1 DSP Virus kit), II (EZ1 Virus mini kit), III (QIAamp DSP virus kit), and IV (heating); the extractions are made from plasma collected on EDTA tubes, and the concentration of extracted DNA was measured on NanoDrop Lite followed by real-time CMVPCR using an Artus CMV QS-RGQ kit. All protocols are performed following the manufacturer’s instructions. **Results**: This study is conducted on the samples of 135 transplant patients whose follow-up medical tests related to human cytomegalovirus infection; since most of the CMVPCR results are negative, we have chosen the 10 CMVPCR positive samples and 2 negative samples as controls to conduct this comparison study. By using NanoDrop Lite to evaluate the DNA concentration, the yield of extracted DNA is higher in our heating protocol than other protocols, the EZ1 DSP virus kit and EZ1 Virus mini kit show homogeneous quantities, and the QIAamp DSP virus kit shows very low DNA yields. Comparing cycle threshold and viral loads by real-time PCR, all these protocols identified negative samples (100%), and the previously positive samples used were as follows: protocol IV (90%), protocol II (60%), and protocol I (40%). QIAamp DSP virus kit results were not real-time PCR applicable and were non-conclusive because of the low DNA yields. **Conclusion**: Our developed heating method (protocol IV) is very effective, reliable, simple, fast, and cheap compared to the other protocols in our study.

## 1. Introduction

Viral opportunistic infections are major causes of morbidity and mortality in solid organ and hematopoietic stem cell transplant recipients [1]. These viral infections in the immunocompromized patients can be particularly difficult to diagnose and treat effectively. The family of the Herpesviridae, such as human cytomegalovirus (HCMV), Epstein–Barr virus (EBV), and BK virus (BKV) are among the most common viral infections after allogeneic transplantation [2]. Particularly, HCMV infection most commonly occurs through reactivation of a latent infection enabled by impaired T-cell-mediated immunity and phagocytic function [3]. Clinical manifestations can range from mild to severe life-threatening diseases [4] and can present as a non-specific systemic viral disease [5] or a localized tissue-invasive infection, most commonly including pneumonia [6], hepatitis [7], colitis [8], and retinitis [9,10]. The diagnostic of human cytomegalovirus plays an important role in the follow-up and treatment of transplant patients because it is a cause of graft rejection [11]. Different studies report the diagnostic methods of human cytomegalovirus, such as the serological methods that determine the presence of IgG antibodies, which is an indicator of infection in the past and of IgM for recent infection [12]. Nowadays, the different standard kits for the diagnosis of CMV are commercialized [13], and it is also possible to diagnose this virus by cell culture on human fibroblasts due to its cytopathic effects [14] characterized by the swelling of infected cells which correlate with the viral load in the clinical sample. Serum is the first biological liquid most used in research and diagnostics to treat hematological, biochemical, and serological diseases [15]; the different diagnostic methods used most are validated on serum as immunofluorescence [16], chemiluminescence [17], immunochromatography [18], ELISA [19], and real-time PCR [20]. The viral load of human cytomegalovirus remains a reference at the clinical stage which enables us to know the progress of treatment of the concerned patients [21]. Using the polymerase chain reaction (PCR) method [20], the quantification of human CMV is realized in the various biological fluids, such as whole blood, plasma [22], white blood cells, biopsies [23], urines [24], cerebrospinal fluid (CSF) [25], and bronchoalveolar fluid [21]. Due to the multitude of different biological fluids [22], the methods of nucleic acid extraction are numerous, and physicians are exposed to a multitude of choices [13,16,17,18,19,20], so it is important to know which method is the most suitable in order to have maximal yield in quality, purity, and nucleic acid concentrations from a given sample type [26]. Another study has reported the advantages of using specific extraction protocols for each type of sample [27]. Extraction protocols can be manual or automatic and are used in scientific research and in vitro diagnostics (IVD). The choice of a method depends on the type of samples, protocol specificity, and extraction time duration. The reliability and efficiency of the different kits are reported in different studies [28,29]. Recently, automatic extraction kits have been used more than manual kits because of the rapid purification and the minimal risk of cross-contamination [30,31,32,33]. We can mention the phenol-chloroform extraction, which uses the principle based on the difference in solubility between nucleic acids and proteins in a two-phase emulsion, and the selective precipitation of nucleic acids in ethanol or isopropanol by centrifugation [34]. Other methods use the principle of silica membrane fixation, where the nucleic acids adsorb in the presence of a high concentration of chaotropic salts which remove water from the hydrated molecules in solution; this method uses the property of DNA to adsorb on the silica membrane and eliminates the rest [35]. With the progress of science and technology, the automatic method that uses the magnetic bead principle is available and saves time where there are a lot of samples [24]. Now, for reasons of reproducibility, those extraction kits are highly recommended; however, they are very expensive. The search for an efficient and less expensive protocol is highly recommended. Therefore, in this study four extraction protocols are compared with a heating protocol to assess the robustness of the findings of each method.

## 2. Materials and Methods

### 2.1. Patients and Samples

This study is conducted on the samples of 135 transplant patients whose follow-up medical tests related to human cytomegalovirus at the AL KINDY laboratory in CASABLANCA-MOROCCO from 2017 to 2021. Most of the CMVPCR results are negative so we have chosen the 10 CMVPCR positive samples and 2 negative samples as controls to conduct this comparative study of the extraction methods. All subjects gave their informed consent for inclusion before they participated in the study.

### 2.2. Methods

In this study, 4 nucleic acid extraction protocols are used according to the manufacturer’s instructions: one manual extraction with the QIAamp DSP Virus kit (Qiagen, Hilden, Germany) (Protocol III), two automatic extractions with EZ1 Advanced XL using the EZ1 DSP virus kit (Protocol I) and the EZ1 Virus mini kit (Qiagen, Hilden, Germany) (Protocol II), and direct detection after the heating of a sample (Protocol IV). The blood of the transplanted patients was collected on EDTA tubes, after centrifugation at 4000 rpm for 10 min, the plasma was collected, and each sample was divided into 4 separate volumes to cover the 4 protocols. The extraction was followed by nucleic acids measured with a NanoDrop Lite spectrophotometer (Thermo Fisher Scientific, Waltham, MA, USA), 0.8% agar’s gel, and amplification of human cytomegalovirus by real-time PCR with an Artus CMV-QS-RGQ kit (Qiagen, Hilden, Germany), and HCMV quantification was compared directly between all the protocols. Each sample was measured 3 times and average values are reported in this study.

## 3. The Sample Pre-Treatment

Whole blood is collected from the patients in the tube containing EDTA as an anticoagulant, this sample is centrifuged at 4000 rpm to recover plasma, and this plasma is used for nucleic acid extraction with the 4 protocols available in the laboratory.

## 4. Nucleic Acids Extraction Methods

### 4.1. Magnetic Bead Automatic Extraction of Nucleic Acids with Ez1 Dsp Virus Kit (Protocol I)/Ez1 Virus Mini Kit (Protocol II)

The nucleic acid extraction steps are performed on the EZ Advanced XL (Qiagen, Hilden, Germany) according to the manufacturer’s instructions. The protocol is available at https://www.qiagen.com/us/products/diagnostics-and-clinical-research/solutions-for-laboratory-developed-tests/ez1-advanced-xl-instrument/ (accessed on 15 August 2020). The purified nucleic acids are eluted and stored in −20 °C pending PCR amplification and agar’s gel, and the manipulation is finished by UV decontamination.

### 4.2. Silica Membrane Manual Extraction of Nucleic Acids with QIAamp DSP Virus Kit (Qiagen, Hilden, Germany)

Manual extraction of nucleic acids is carried out according to the manufacturer’s protocol using the principle of silica membranes. The protocol is available at https://www.qiagen.com/us/shop//sample-technologies/dna/qiaamp-dna-mini-kit/#resources (accessed on 15 August 2021).

### 4.3. Direct Extraction by Heating of Nucleic Acids

This method is used without recourse to kits, we took 90 µL of plasma and incubated it with protease Q (Qiagen, Hilden, Germany) for 15 min at 56 °C and then inactivation at 90 °C for 10 min; we then centrifuged and collected the supernatant that will be used immediately for CMVPCR amplification of human cytomegalovirus.

## 5. Measurement of Nucleic Acid Concentration and Quality

To assess the quantity and quality of the extracted viral DNA, the absorbance at 260 nm of all samples was measured by a NanoDrop Lite spectrophotometer (Thermo Fisher Scientific, Waltham, MA, USA). The absorbance ratio 260/280 for pure DNA free of protein was reported ~1.8. Each sample was measured 3 times and average values are reported in this study.

## 6. Amplification of Human Cytomegalovirus Genome with Artus CMV-QS-RGQ

The extracted DNA is amplified and quantified using the Artus CMV-QS-RGQ kit (Qiagen, Hilden, Germany). In each PCR tube, the reaction mixture is composed of 12.5 µL of the master mix, 2.5 µL of 100 mM MgSO_4_, and 10 µL of extracted DNA. The quantification of the viral load is performed using the curve drawn from the 4 standards, which are QS1 (10,000 copies/µL), QS2 (1000 copies/µL), QS3 (100 copies/µL), and QS4 (10 copies/µL). The results are displayed as an amplification curve with a cut-off that shows how the gene of interest uses the reaction mixture as the cycle progress. The 109 min thermal cycler program was as follows: the pre-denaturation step of 10 min at 95 °C, followed by 45 cycles of 15 s at 94 °C of denaturation, 30 s at 65 °C of annealing, and 20 s at 72 °C of extension. The quantification of the internal control is performed by detecting its fluorescence in the yellow channel at 560 nm and the cytomegalovirus gene is performed in the green channel at 530 nm. The quantity can be converted from copies/µL and copies/mL or IU/mL, taking into account the starting sample volume and elution volume. The analysis parameters to validate the protocol are the following: the threshold is fixed at 0.03, the presence of a dynamics tube, and no correct slope. To validate our hypothesis the QS must have the value of Ct, R2 > 0.98, the slope M (−3 and −3.6), the negative sample must have no Ct, and the positive sample must have a Ct value.

## 7. Statistical Data Analysis

**Figure 1:**
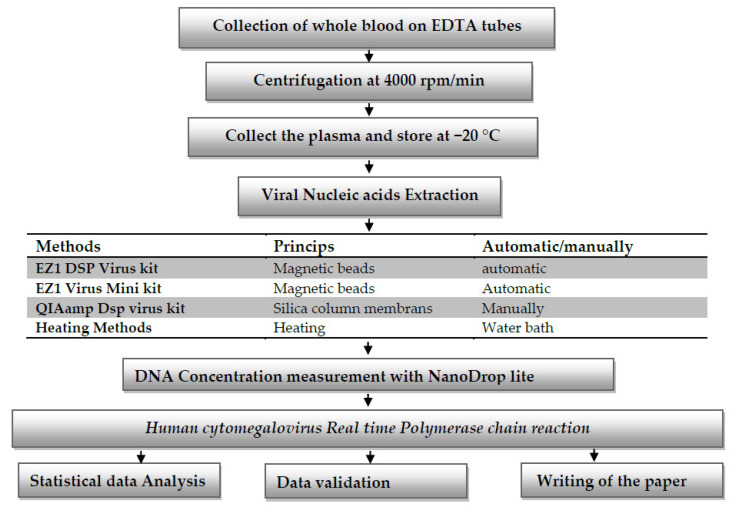
Expermental design that covers the work conducted in our study.

All statistical analyses were performed using Microsoft Excel data analysis, normality was tested for all datasets using the D’Agostino Pearson omnibus normality test (Shown in Figure 1). The Kruskal–Wallis test with Dunn’s correction and the Mann–Whitney test were conducted to compare the yield of nucleic acids. Mean values of statistical data and standard deviation curves were calculated using Grubbs tests on the 12 samples used and extracted 3 times for each protocol. Statistically significant values were considered to be *p* < 0.05. (https://www.graphpad.com/quickcalcs/Grubbs1.cfm, accessed on 2 June 2021), a *p*-value of less than 0.05 was considered statistically significant. 

## 8. Results

### 8.1. Yield and Quality of Extracted Nucleic Acids

**Figure 2:**
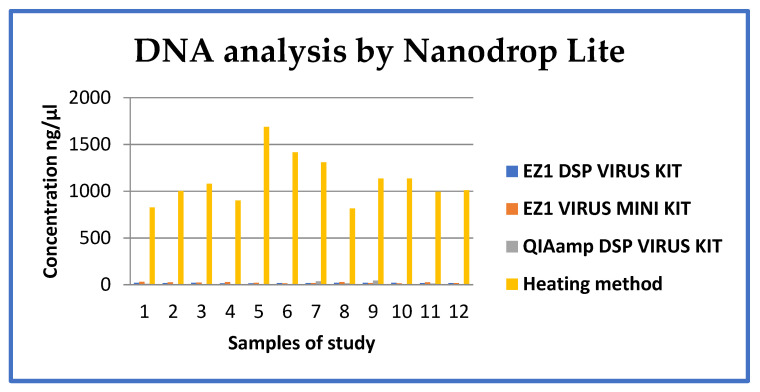
Graph of nucleic acid quantification using NanoDrop Lite.

The concentration and quality of extracted nucleic acids were compared using the four DNA extraction protocols. Average nucleic acid concentrations in elutions from each method of 14.35–20.52 ng/µL, 13.18–28.92 ng/µL, 0.3–42.40 ng/µL, and 815.76–1687, 63 ng/µL were obtained with the EZ1 DSP Virus kit, EZ1 Virus mini kit, QIAamp DSP Virus kit and heating protocol, respectively (Figure 2 and Table 1). The nucleic acid concentration was significantly higher with the heating protocol than the three other protocols (*p* < 0.0001). There was a statistically significant difference in nucleic acid concentration between automatic and manual methods (*p* = 0.5581), and no significant difference in nucleic acid concentration between the two automated protocols of the EZ1 (*p* = 0.5746).

### 8.2. Nucleic Acid Extraction and Real-Time PCR Amplification

When extracting nucleic acids from 200 µL of blood plasma and 60 µL of elution, the automatic methods using the EZ1 DSP virus kit and EZ1 virus mini kit show the same sensitivity with almost equal nucleic acid concentrations as shown in this study. The manual method using the QIAamp DSP Virus kit had low efficiency with low nucleic acid concentrations; this may be caused by the losses incurred during manual handling and ethanol 96% cross-contamination risk. The new heating method developed in this study showed interesting results with high nucleic acid concentrations from 90 µL, as shown in the results. The detection of human cytomegalovirus by the artus CMV QS-RGQ kit (Qiagen, Hilden, Germany) showed the high sensitivity and efficiency of our heating method compared to the other protocols because out of the 10 samples already positive to HCMV, all were positive, which means 90% were the same, while the 2 negative samples were 100% negative. The two automatic protocols using the EZ1 DSP virus kit and EZ1 virus mini kit show different sensitivities (40 and 60%, respectively) on the positive samples. As the EZ1 DSP Virus kit is the main kit used in the laboratory, two samples that were negative (while positive with the heating protocol) were redone by increasing the volume of extract from 10 µL to 20 µL, the results became positive which explains the sensitivity of the EZ1 DSP Virus kit and a recommendation to use 20 µL of extract as suggested by the manufacturer protocol.

### 8.3. Nucleic Acid Concentration and Quality Assay by NanoDrop Lite

Each sample was divided into four separate volumes to cover the four protocols, 200 µL was used in protocols I, II, and III, and 90 µL was used for protocol IV (heating). Using a NanoDrop assay, each sample was measured three times and the average values are reported in Table 1 and Figure 1. Results show that the heating protocol (IV) results in a high nucleic acid concentration, followed by protocols I (EZ1 DSP Virus kit) and II (EZ1 Virus Mini kit), and the QIAamp DSP virus kit (protocol III) resulting in very low concentrations.

### 8.4. Effect of Sample Incubation in Protocol IV

Protocol IV consisted of heat samples at 56 °C for 10 min followed by 90 °C for 15 min, after centrifugation the supernatant was measured, and the results in ng/µL are in Table 1. This protocol shows a very significant increase in DNA yield and confirms the recommendation of different researchers to consider this protocol as a possibility but that it still requires many studies to validate the different biological samples.

### 8.5. Comparison of the Viral Loads of the Different Protocols

Ten previously confirmed positive (CMV+) and two negative (CMV−) samples were used, results were measured in copies/mL, IU/mL, and log IU/mL to facilitate clinical interpretation. Ct values (cycle threshold) showed that the smaller the value, the higher the viral load and that it is recommended to use Ct values to interpret the results. We evaluated and compared the Ct values and viral load of three protocols according to the manufacturer’s instructions, all three protocols gave the expected results for the two negative samples. Regarding the 10 positive samples, the heating protocols, EZ1 DSP virus kit, and EZ1 Virus mini kit resulted in 9/10 (90%), 4/10 (40%), and 6/10 (60%), respectively (Table 2). Sample 2 (CMV+) of the EZ1 DSP virus kit protocol became negative, while it is positive in other protocols (EZ1 Virus mini kit and heating). We decided to redo the real-time PCR using 20 µL with the EZ1 DSP virus kit, and we found a positive result (Ct: 25.650 and viral load of 16,543.323 IU/mL). This result is proportional to the result of the EZ1 virus mini kit (Ct: 25.610 and viral load of 19,574.810 IU/mL), and this is proof of the role played by the eluent volume in results interpretation. Despite the elimination of the QIAamp DSP virus kit DNA yield due to the low DNA yield < 10 ng/µL which is a non-PCR applicable value, sample four and eight (>10 ng/µL) from the QIAamp DSP virus kit were used to quantify CMV viral load. Sample four became positive like the other protocols used in this study, while sample eight became negative. We concluded that the causes of low efficiency with low DNA yield may be the losses and the 96% ethanol cross-contamination risk incurred during manual handling.

We used aliquots of DNA that had been stored at −20 °C for more than 6 and 12 months to identify whether the length of storage time could affect the real-time PCR results. We performed the real-time PCR in duplicate and the values were unchanged, and we have concluded that there is no effect of the storage time on DNA yield and real-time PCR viral loads results. According to the manufacturer’s instructions, the RNA carriers included in the kit are used to protect against DNA degradation. On the other hand, the extracts in the heating method cannot be stored because the enzymes degrade the nucleic acids, and the conservation will not be able to give expected results. As a result, this heating method is recommended for discovering PCR amplification immediately.

### 8.6. Reproducibility of Values, Eluent Volume Effect, Checking with QIAamp DSP Virus Kit

In our study a positive sample was performed in duplicate to ensure the values of real-time PCR amplification; the results of the Ct (Cycle of threshold) and viral load are 25.71 with a viral load of 15,417.3 IU/mL, and 25.45 with a viral load of 18,373.07 IU/mL (Table 3). This repeatability is proof of reliability on all the results found in this study. The sensitivity of the EZ1 DSP virus kit is confirmed by increasing the eluent volume from 10 µL to 20 µL; at 10 µL the two samples were negative but became positive at 20 µL, which was the reason for this sensitivity (Table 4). When testing the results of the QIAamp Virus kit, we did not have any conclusive results to report because the DNA yield was <10 ng/µL, which is not PCR applicable.

### 8.7. Comparison of Different Kits and Method Parameters

Recapitulating the various parameters, we compared in our study, based on the different results we found, the heating method appears to be simple, rapid, efficient, and cost-effective (Table 5).

## 9. Discussion

The experiments performed in this study were carried out in a platform laboratory equipped with the kits and machines from QIAGEN: EZ1 DSP virus kit, EZ1 Virus mini kit, QIAamp DSP virus kit, and EZ1 Advanced XL, Artus CMV-QS-RGQ and ROTOR Gene thermocycler. The reliability and sensitivity of the kits used in our studies are already reported in the bibliography. The EZ1 virus kit uses magnetic bead technology to extract viral and bacterial nucleic acids from samples such as serum and plasma [22], cerebrospinal fluid (CSF) [25,36,37], urine [19,24], whole blood [38,39], stool [40], transport medium [41], and respiratory samples [42]. It is a 1 h automated process and it can be used for PCR, real-time PCR, and genotyping application. We thought of conducting this comparison ourselves to check the difference between the extraction principles used, such as the magnetic beads, silica membrane, and home-made heating method, even if these kinds of studies already exist in the bibliography. This article and those that will follow are a message to researchers that there are opportunities to conduct research with kits and materials that we have at hand.

The molecular biology platform is not cost-free and the real-time PCR tests are expensive. The EZ1 DSP Virus kit package costs $475.00 for 48 tests, and the EZ1 Virus Mini kit costs $443.00 for a 48-test kit. The QIAamp DSP virus kit costs $365.00 for 50 tests (Table 5); this is a kit that uses the silica membrane principle, and this can be used automatically on the QIAcube or manually. Automatic extractions give more easily reproducible, efficient, and accurate results than manual extractions, and amplification becomes efficient, more sensitive, specific, and without cross-reactions [43]. The manual QIAamp DSP virus kit used in our study did not give significant results, and values were not very homogeneous; this may be due to ethanol used in the extraction steps which may cause contamination. Our case does not confirm the reliability of the QIAamp DSP virus kit reported in previous studies [44]. We still notice the inconvenience of false negatives or false positives which the QIAamp DSP virus kit is proof of. This diagnostic mistake has shown us that it is required to have at least two extraction methods in a laboratory, one to confirm the results of the other, especially if the results of the first method are not in accordance with the patient clinical symptoms. This will reduce the risk of providing unreliable results to patients.

Quantification remains an essential tool for monitoring and treating patients by predicting the result of treatments based on the viral load in the sample and will depend on the used methods [13,15,19,20,45]. The kit’s non-specificity of sample type can cause the loss of nucleic acids and low quantification [33]. We have noticed that manual protocols have low detection limits compared to automatic protocols [46]. False results, especially negatives that become positive with other manual methods, are explained by handling more steps, and the more samples, the more contamination occurs [47]. The total nucleic acid extracts using the four protocols show that the yield of the QIAamp DSP virus kit is lower than that of the EZ1 DSP virus kit and EZI virus mini kit. With the arrival of nucleic acid extraction kits on the market, this heating method which is a basic method has been overlooked even though it has many advantages. However, nowadays, most of the extraction kits on the market integrate the heating procedure, even if each kit with different reagents is available in the kit. We have brought it back to encourage researchers to evaluate it on different types of samples [22,24,25] so that we can conclude its effectiveness.

Several studies report the advantage of this heating method compared with other extraction methods. Reischl et al. reported similar PCR results by heating enzymatic extractions [48]. Evaluating the efficiency of the phenol/chloroform method, heating method, and QIAamp DSP virus kit, Chan et al. noted a correlation of real-time PCR results from these three methods and confirmed the place and advantage of the heating protocol as a less expensive method [49]. The heating protocol is reported to be a time-saving method, but more studies are needed to evaluate this method on all biological samples, such as plasma or serum [22], CSF [25], and urine [24], and to establish a standard protocol with optimal steps and conditions regarding the different pathogens, such as viruses, bacteria, parasites, and fungi. Studies reported that the heating protocol can detect the E gene of SARS-CoV-2 in nasopharyngeal swabs, and it showed interesting results with a specificity of 100% and sensitivity of 79% [50]. All studies prove that the combination of a heating protocol and isothermal amplification is more economical and time saving than other current methods. The use of kits on automatic Biorobots has revolutionized extraction methods, and different studies report no statistically significant difference between the EZ1 DSP virus kit and EZ1 virus mini kit. The protocols used in this study are all validated and are on the market for use; although the different studies show the effectiveness of the QIAamp DSP virus kit, in our study this kit did not give interpretable values. The heating method gave the expected results which confirm why most extraction kits nowadays add in the instructions for use, for the possibility to use it. It is recommended to check the specificity between types of samples [22,24,25] and the kit, and it is necessary to follow the manufacturer instructions carefully to avoid false results. This study recognized the limitations that will be taken into consideration in the following research, that is: the small number of samples, the types of samples, the methods used, and patient’s type of pathology. The heating method developed in our study shows reliable results in accordance with those of the real-time PCR used as controls. To guarantee the reliability of a heating method, we suggest increasing the number of samples; integrating various types of samples such as cerebrospinal fluid (CSF), urine, or nasopharyngeal swabs; comparing this method with other methods such as cell culture or ELISA; and expanding various patients’ pathologies related to human cytomegalovirus infection, such as pneumonia, hepatitis, colitis, and retinitis. These four points will improve the confidence in this method which is often overlooked in research dominated by the use of kits. With that, we decided to develop our protocol and we wish to standardize it as they give promising results.

## 10. Conclusions

Given the many extraction and amplification methods on the market, the specificity between the sample type and the kit should be carefully checked to avoid false results. Each laboratory should have more than one extraction method to ensure the reliability of the results. The correlation between clinical symptoms and patient history is an important factor to check and the viral loads resulting from real-time PCR amplification will guide the follow-up and treatment of patients. The results show that protocol I is very sensitive to the eluent volume change. In fact, samples that are previously positive become negative depending on eluent quantity in the real-time PCR reaction mixtures, and vice versa. Method IV has not shown any variation in results, and it is a simple, fast, and cheap protocol with promising results. We recommend this method without the need for extraction kits, and we suggest further research to use and verify it on different types of samples to confirm this alternative method. Our study is conducted on a small sample group; our priority in the future is to repeat this study with larger sample numbers to check if there will be a significantly different conclusion to this study. We encourage research on human cytomegalovirus in other fluids such as saliva, pleural fluid, synovial fluid, puncture fluid, joint fluid, bronchial fluid, ascites fluid, salivary fluid, bronchoalveolar fluid, and also in feces. 

In future studies it is recommended to increase the number of samples, the number of protocols and to vary the different biological fluids and sample volumes.

## Figures and Tables

**Table 1:** Summarized table of viral DNA concentrations for the four protocols used in our study.

Samples	Status (CMVPCR)	EZ1 DSP Virus Kit (I) [C] ng/µL ± SD	EZ1 Virus Mini Kit (II) [C] ng/µL ± SD	QIAamp DSP Virus Kit (III) [C] ng/µL ± SD	Heating (ng/µL) (IV) [C] ng/µL ± SD
S1	+	18.38 ± 0.1979	28.92 ± 1.4600	8.05 ± 0.1898	826.7 ± 1.110
S2	+	17.23 ± 0.4213	24.38 ± 0.6099	0.73 ± 0.0163	1001.6 ± 1.420
S3	+	20.52 ± 1.3501	22.47 ± 0.2523	0.3 ± 0.0472	1079.7 ± 1.118
S4	+	15.45 ± 1.3797	27.98 ± 1.2840	2.6 ± 0.5818	901.8 ± 0.815
S5	+	14.35 ± 1.9719	19.03 ± 0.3918	3.13 ± 0.5437	1687.6 ± 2.266
S6	+	18.08 ± 0.0363	13.18 ± 1.4872	0.75 ± 0.0148	1416.9 ± 1.205
S7	+	17.08 ± 0.5021	17.03 ± 0.7663	35.05 ± 1.7523	1310.2 ± 0.786
S8	+	19.63 ± 0.8709	27.03 ± 1.1061	14.38 ± 0.2655	815.8 ± 1.531
S9	+	20.47 ± 1.3232	16.10 ± 0.9404	42.4 ± 2.2810	1135.6 ± 1.101
S10	+	19.18 ± 0.6286	15.50 ± 1.0528	3.8 ± 0.4955	1135.6 ± 1.451
S11	-	17.95 ± 0.0337	23.88 ± 0.5163	11.8 ± 0.0800	994.8 ± 1.455
S12	-	17.83 ± 0.0983	17.97 ± 0.5903	5.27 ± 0.3897	1010.4 ± 1.390

**Table 2:** Human cytomegalovirus viral loads obtained by real-time PCR quantification using the Artus CMV QS-RGQ kit.

Samples	EZ1 DSP Virus Kit (I)		EZ1 Virus Mini Kit (II)		Heating (ng/µL) (IV)	
#	Ct	Viral Loads (IU/mL)	Ct	Viral Loads (IU/mL)	Ct	Viral Loads (IU/mL)
1	-	-	-	-	25,420	65,338,158
2	-	-	25,610	32,102,688	30,730	1,507,554
3	-	-	29,810	1,630,111	36,630	22,780
4	23.64	230,604,730	23,160	183,010,913	25,420	65,089,501
5	30,200	2,190,564	31,300	564,767	26,560	29,003,613
6	35,400	44,050	36,130	26,125	23,410	217,982,896
7	31,360	772,030	30,610	1,316,641	40,620	2722
8	-	-	-	-	25,070	67,487,230
9	-	-	-	-	-	-
10	-	-	-	-	38,160	18,302
11	-	-	-	-	-	-
12	-	-	-	-	-	-

**Table 3:** Repeatability proven using sample 4, which took away the doubt on all the results used in this study.

Samples	EZ1 DSP Virus Kit (I)		Results
#	Ct	Viral loads (IU/mL)	
Sample 4	25.71	15,417.3 IU/mL	Positive
	25.45	18,373.07 IU/mL	Positive

**Table 4:** The sensitivity of the DSP virus kit is confirmed by increasing the volume of eluent from 10 µL to 20 µL, the two samples that were negative at 10 µL became positive at 20 µL, which was the reason of this high sensitivity of volume.

Samples	EZ1 DSP Virus Kit (I)		Results
#	Ct	Viral Loads (IU/mL)	
1 (10 µL) study value	-	-	Negative
1 (20 µL) reference validated value	25.61	19,574.810 IU/mL	Positive
1 (20 µL) study value	25.65	16,543.323 IU/mL	Positive

**Table 5:** Comparison of different kits and method parameters. N.A: Not applicable.

Parameters	Kits/Method				Comments
	EZ1 DSP Virus	EZ1 Virus Mini	QIAamp DSP Virus	Heating Method (Self-Made)	
Costs/kit or test ($)	475 $/48 tests	443 $/48 tests	365 $/50 tests	Not on the market 3.00 $/test	Heating method is cheap and simple
Cost/test ($)	9.89 $	9.23 $	7.3 $	3.00 $	
Required instruments	EZ1 Advanced XL	EZ1 Advanced XL	QIAcube	Water bath	
Extraction duration (hours)	1 h	1 h	1 h	<30 min	Heating method is fast
Positive percentage (%)	40	60	N.A.	90	Heating method is highly sensitive and efficient
Negative percentage (%)	100	100	100	100	
Mean Concentration (ng/µL) ± SD	18.13 ± 0.497	21.12 ± 0.492	10.63 ± 0.511	1109.72 ± 1.500	

## Data Availability

The authors confirm that the data supporting the findings of this study are available from the corresponding author on reasonable request.
